# MUC1 stimulates EGFR expression and function in endometrial cancer

**DOI:** 10.18632/oncotarget.8743

**Published:** 2016-04-15

**Authors:** Brian J. Engel, Jessica L. Bowser, Russell R. Broaddus, Daniel D. Carson

**Affiliations:** ^1^ Department of BioSciences, Rice University, Houston, TX 77005, USA; ^2^ Department of Translational Molecular Pathology, University of Texas MD Anderson Cancer Center, Houston, TX 77030, USA; ^3^ Department of Pathology, University of Texas MD Anderson Cancer Center, Houston, TX 77030, USA; ^4^ Department of Genetics, University of Texas MD Anderson Cancer Center, Houston, TX 77030, USA

**Keywords:** MUC1, EGFR, endometrial cancer

## Abstract

The current standard of care for endometrial cancer patients involves hysterectomy with adjuvant radiation and chemotherapy, with no effective treatment for advanced and metastatic disease. MUC1 is a large, heavily glycosylated transmembrane protein that lubricates and protects cell surfaces and increases cellular signaling through the epidermal growth factor receptor (EGFR). We show for the first time that MUC1 stimulates EGFR expression and function in endometrial cancer. siRNA knockdown and CRISPR/Cas knockout of *MUC1* reduced EGFR gene expression, mRNA, protein levels and signaling. MUC1 bound strongly to two regions of the *EGFR* promoter: −627/−511 and −172/−64. MUC1 knockout also reduced EGFR-dependent proliferation in two dimensional culture, as well as growth and survival in three dimensional spheroid cultures. MUC1 knockout cells were more sensitive to the EGFR inhibitor, lapatinib. Finally, MUC1 and EGFR co-expression was associated with increased cellular proliferation in human endometrial tumors. These data demonstrate the importance of MUC1-driven EGFR expression and signaling and suggest dual-targeted therapies may provide improved response for endometrial tumors.

## INTRODUCTION

The current standard of care for endometrial cancer patients is hysterectomy with adjuvant radiation and chemotherapy. There remains, however, poor survival in advanced disease [1]. Better understanding of cellular mechanisms associated with advanced disease may elicit more effective treatment strategies. MUC1 is a large, heavily O-glycosylated transmembrane protein of epithelial cells, normally providing lubrication and barrier functions [2]. MUC1 consists of two domains: the ectodomain, primarily a variable number of 20 amino acid tandem repeats, and the 158 amino acid C-terminal domain (MUC1-Cter) [3]. MUC1-Cter increases cell signaling and gene expression through cellular receptors, resulting in cell survival, growth, differentiation, and migration [4–10]. MUC1-Cter contains a CQCRRK motif that is necessary for nuclear localization of MUC1 and regulation of gene expression [11]. Despite extensive study of MUC1 in the context of breast and pancreatic cancers, there is limited understanding of MUC1 in endometrial cancer beyond expression studies [12, 13].

EGFR is a receptor tyrosine kinase that drives cellular processes including proliferation, migration and survival. EGFR signals through the MAPK, PI3K, JAK and PLCγ pathways [14]. EGFR expression is increased in endometrial cancer is associated with poor prognosis [15]. MUC1 is known to increase the levels and signaling of EGFR in some cellular contexts [6, 16, 17]; however, the underlying mechanisms are poorly understood. In addition, activation of EGFR is associated with increased levels of MUC1 [18]. The combined influence of MUC1 and EGFR has not been studied in endometrial cancer and any physiological or clinical relevance of their co-regulation is not known.

This study investigates the mechanism and functional consequences of MUC1 driven EGFR expression and signaling in endometrial cancer. We observed indications that MUC1 driven EGFR regulation occurs in endometrial cancers and is likely manifest at two levels: 1) by elevating EGFR levels transcriptionally and; 2) by enhancing EGFR signaling. It is possible that MUC1 and EGFR targeted co-therapies may provide a new avenue for the treatment of advanced endometrial cancer.

## RESULTS

### MUC1 increases EGFR levels in endometrial cancer cells

**Figure 1:**
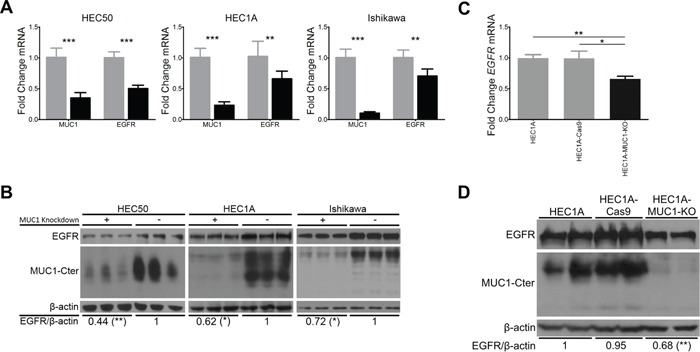
MUC1 increases EGFR mRNA and protein levels in endometrial cancer cell lines **A.** HEC50, HEC1A and Ishikawa cells pretreated with MUC1-targeted siRNA (siRNA-MUC1, black bars) or scrambled siRNA (scRNA, grey bars) and were subjected to qRT-PCR analysis for *MUC1*, *EGFR* and *ACTB* mRNA. Relative levels were normalized to values obtained for scRNA in each case (n=6). **B.** Western blot analysis of HEC50, HEC1A and Ishikawa cells pretreated with MUC1-targeted (+) or scrambled (−) siRNA for EGFR, MUC1-Cter and β-actin. Numerical values represent mean band intensity of EGFR relative to β-actin and then normalized to scRNA (n=6). **C.** qRT-PCR for *EGFR* mRNA levels in MUC1 knockout cell lines. Relative levels were normalized to HEC1A (n=3). **D.** Western blotting of biological replicates for EGFR, MUC1 and β-actin in HEC1A, HEC1A-Cas9 and HEC1A-MUC1-KO cell lines. Numerical values represent mean band intensity of EGFR relative to β-actin normalized to HEC1A (n=2). Student's t-test: * p<0.05, ** p<0.01, *** p<0.001 compared to scRNA (panels A and B) or parental cell line (panels C and D).

To determine the effect of MUC1 on EGFR levels in endometrial cancer, HEC1A, HEC50 and Ishikawa cell lines were treated with *MUC1*-targeted siRNA (siRNA-*MUC1*) or scrambled siRNA (scRNA) control. As expected, siRNA-*MUC1* reduced *MUC1* mRNA 70-90% compared to scRNA control. This was associated with a 30-50% decrease in *EGFR* mRNA (Figure 1A). Western blotting and densitometry showed similar reductions in EGFR protein (Figure 1B). Additionally, CRISPR/Cas gene editing was used to knockout MUC1 expression in the HEC1A cell line (HEC1A-MUC1-KO). HEC1A stably expressing Cas9 (HEC1A-Cas9) maintained MUC1 and EGFR expression, whereas HEC1A-MUC1-KO showed no MUC1 expression and a similar reduction of EGFR expression at the mRNA (Figure 1C) and protein (Figure 1D) levels as *MUC1* knockdown. These data demonstrate that MUC1 increases EGFR mRNA and protein levels.

### MUC1 increases EGFR proximal promoter activity

**Figure 2:**
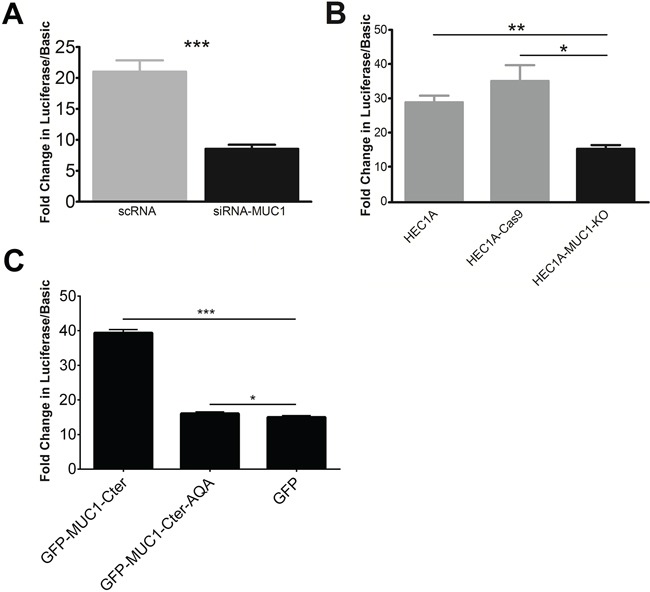
MUC1 stimulates EGFR promoter activity A construct comprised of the 1.1 kb *EGFR* proximal promoter fused to luciferase was used to assess promoter activity. **A.** EGFR promoter activity in HEC50 cells pretreated with either MUC1-targeted (siRNA-MUC1; black bar) or scrambled siRNA (scRNA; grey bar). **B.** *EGFR* promoter activity in HEC1A-MUC1-KO cells compared to HEC1A and HEC1A-Cas9 cells. **C.** EGFR promoter activity of HEC50 cells transiently transfected with GFP-tagged MUC1-Cter (GFP-MUC1-Cter), GFP-tagged MUC1 nuclear localization mutant (GFP-MUC1-Cter-AQA) or GFP alone. Student's t-test: * p<0.05, ** p<0.01, *** p<0.001 compared to scRNA (panel A), HEC1A-MUC1-KO (panel B) or GFP (panel C).

We next tested the effects of MUC1 on *EGFR* gene expression using a luciferase expression vector driven by the −1109/−1 region of the *EGFR* promoter. Treatment of HEC50 cells with siRNA-*MUC1* decreased EGFR promoter activity by 60% compared to scRNA control (Figure 2A). A similar reduction was observed in HEC1A-MUC1-KO cells compared to HEC1A and HEC1A-Cas9 (Figure 2B). In addition, overexpression of GFP-tagged MUC1-Cter (GFP-MUC1-Cter) in HEC50 cells increased *EGFR* promoter activity (Figure 2C). Mutation of CQC to AQA (GFP-MUC1-Cter-AQA) abrogates this effect. Thus, MUC1 increases activity of the *EGFR* proximal promoter.

### MUC1-Cter binds to the EGFR promoter

**Figure 3:**
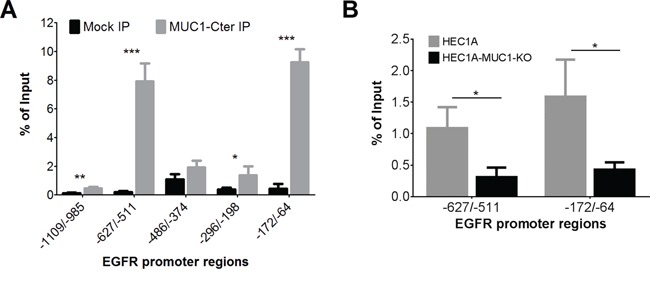
MUC1 binds to the −627/−511 and −172/−64 regions of the EGFR promoter **A.** ChIP analysis of HEC50 chromatin enriched with a no antibody control (black bars) or a MUC1-Cter-directed antibody (grey bars) *via* qRT-PCR of the indicated regions of the *EGFR* promoter. **B.** ChIP analysis for MUC1-Cter in HEC1A and HEC1A-MUC1-KO cells. Student's T-test p-values ≤0.05 (*), ≤0.01 (**), ≤0.001 (***), n=3.

We next considered that increased *EGFR* gene expression occurs through direct interaction with MUC1-Cter. MUC1-Cter-directed ChIP of HEC50 chromatin showed enrichment of MUC1-Cter in the *EGFR* promoter regions −1109/−985, −627/−511, −486/−374, −296/−198 and −172/−64 as compared to a mock ChIP control. The highest enrichment was observed in the −627/−511 and −172/−64 regions (Figure 3A). Enrichment of the −627/−511 and −172/−64 regions was confirmed in HEC1A cells *vs* HEC1A-MUC1-KO (Figure 3B) indicating that direct interaction of MUC1-Cter increases expression of the *EGFR* gene. Transcription factor binding site analysis with ALGGEN-PROMO 3.0 identified CCAAT/enhancer binding protein β, p53 and glucocorticoid receptor α as putative MUC1-Cter binding partners in these regions (data not shown).

### MUC1 increases EGF-dependent signaling, cellular proliferation and spheroid survival

**Figure 4:**
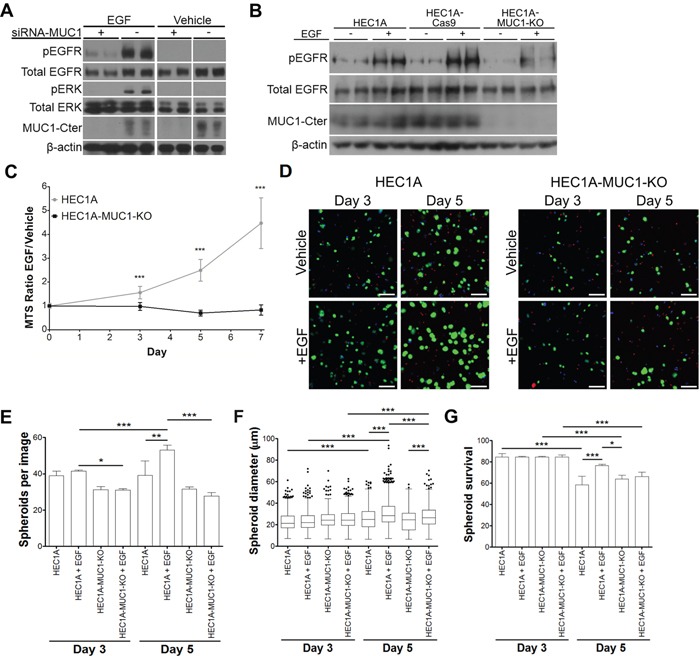
MUC1 stimulates EGFR signaling, cell proliferation and 3D spheroid survival **A.** Western blotting of duplicate HEC50 cells pretreated with MUC1-targeted (+) or scrambled (−) siRNA followed by 5 min treatment with 10 ng/mL EGF or vehicle. **B.** Western blotting of duplicate samples of HEC1A, HEC1A-Cas9 and HEC1A-MUC1-KO lines treated with 10 ng/mL EGF (+) or vehicle (−) for 5 min. **C.** MTS assay of HEC1A and HEC1A-MUC1-KO lines incubated in serum-free medium with 50 ng/mL EGF or vehicle (0.1% BSA in PBS). MTS absorbance is reported as the ratio of EGF treatment to vehicle. **D.** Representative images of 3D spheroids stained for living cells (green), dead cells (red) and nuclei (blue). Scale bars represent 50 μm. **E.** EGF-dependent spheroid frequency. Bars represent means + standard deviations per field obtained from at least 7 different fields in each case. **F.** EGF-dependent effects on spheroid diameter. Data represented as Tukey box plots from at least 650 spheroids in each case. **G.** EGF effects on spheroid survival. Statistics: *, p<0.05; **, p<0.01; ***, p<0.001 as determined by Student's T-test (panel C) or ANOVA followed by Tukey's post-hoc test (panels E, F, G).

To test the effect of MUC1 on EGFR signaling, HEC50 cells were serum starved and then treated with EGF for 5 min. Western blotting showed a marked decrease in phosphorylation of EGFR (pEGFR) and ERK (pERK) in siRNA-*MUC1* treated cells compared to scRNA (Figure 4A). Similarly, EGF-stimulated HEC1A-MUC1-KO cells had decreased pEGFR compared to HEC1A and HEC1A-Cas9 cells (Figure 4B). Reduced EGFR activation in the absence of MUC1 could be a result of EGFR localization. Immunostaining of siRNA-*MUC1* treated HEC50 or HEC1A-MUC1-KO cells showed punctate EGFR, indicative of intracellular localization (Supplementary Figure 1).

To evaluate the physiological effect of MUC1-dependent EGFR signaling, cells were incubated in serum free media with 50 ng/mL EGF or vehicle (0.1% BSA in PBS). HEC1A cells showed increased growth in the presence of EGF, whereas HEC1A-MUC1-KO cells showed no increase (Figure 4C). HEC1A and HEC1A-MUC1-KO cells were encapsulated into hyaluronic acid-based hydrogels in the presence of serum free media with 50 ng/mL EGF or vehicle and stained for living cells, dead cells and nuclei after 3 or 5 days (Figure 4D). CellProfiler image analysis was used to calculate spheroids per field (Figure 4E), spheroid diameter (Figure 4F), and spheroid survival (Figure 4G). There was an increase in HEC1A spheroids per field in the presence of EGF, whereas HEC1A-MUC1-KO spheroids showed no change. HEC1A spheroids showed a more robust increase in diameter from EGF treatment than the HEC1A-MUC1-KO spheroids. While all spheroids had similar survival at day 3, EGF treated HEC1A spheroids on day 5 showed significantly higher survival than all other conditions. Collectively, these data demonstrate that MUC1 increases EGFR signaling, proliferation, spheroid formation and survival.

### MUC1 knockout sensitizes cells to the EGFR inhibitor, lapatinib

**Figure 5:**
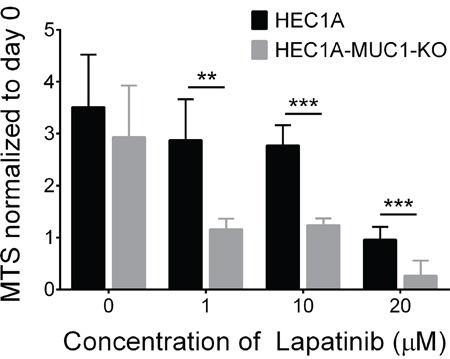
MUC1 knockout sensitizes cells to the EGFR inhibitor, lapatinib HEC1A and HEC1A-MUC1-KO cells were seeded into a 96 well plate and grown for 24 hours. Subsequently, cells were treated for 72 hours with 1, 10 or 20 μM lapatinib in DMSO or DMSO alone followed by MTS assay. MTS assay absorbance after drug treatment was normalized to day 0 values. Values represent five independent biological replicates in each case. Student's t-test: ** p<0.01, *** p<0.001.

We considered that MUC1 knockout may sensitize cells to EGFR inhibitors. To test this, HEC1A and HEC1A-MUC1-KO cells were treated with erlotinib, AG490 or lapatinib followed by MTS assays. Both cell lines were resistant to erlotinib and AG490 at concentrations up to 20 μM (data not shown). HEC1A-MUC1-KO cells, however, had significantly reduced MTS absorbance at 1, 10 and 20 μM lapatinib *vs* HEC1A (Figure 5). These data suggest that coupling MUC1 suppression with lapatinib treatment provides more therapeutic benefit than EGFR inhibition alone.

### MUC1-EGFR co-expression is associated with higher cellular proliferation in human endometrial tumors

**Figure 6:**
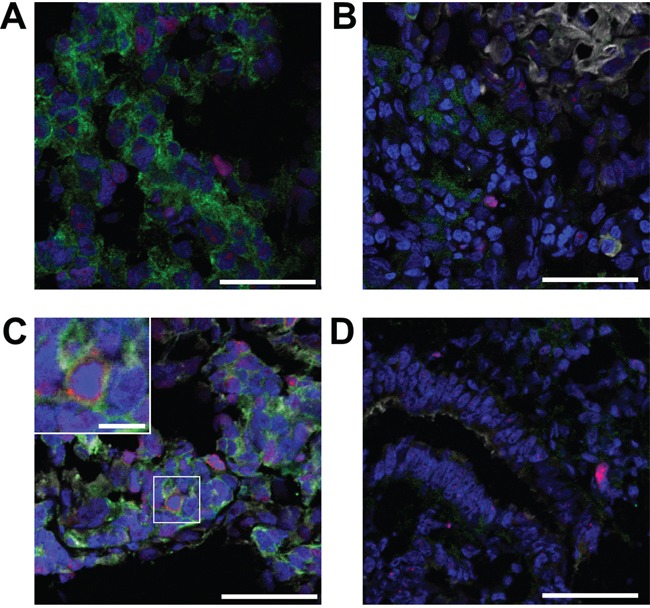
Representative MUC1 and EGFR staining patterns observed in human tumors Endometrial tumor sections were stained for nuclei (DAPI, blue), EGFR (green), Ki67 (red) or MUC1 (white). **A.** Malignant mixed Mullerian tumor (MMMT) with EGFR staining, but lacking MUC1. **B.** Grade 1 tumor with MUC1 and EGFR staining, but little co-expression. **C.** MMMT tumor with high MUC1-EGFR co-expression. Regions with MUC1 and EGFR co-expression have elevated Ki67 staining (inset). **D.** Normal uterine tissue with apical epithelial MUC1 expression and low stromal EGFR expression. Scale bars represent 50 μm, inset scale bar 10 μm.

**Figure 7:**
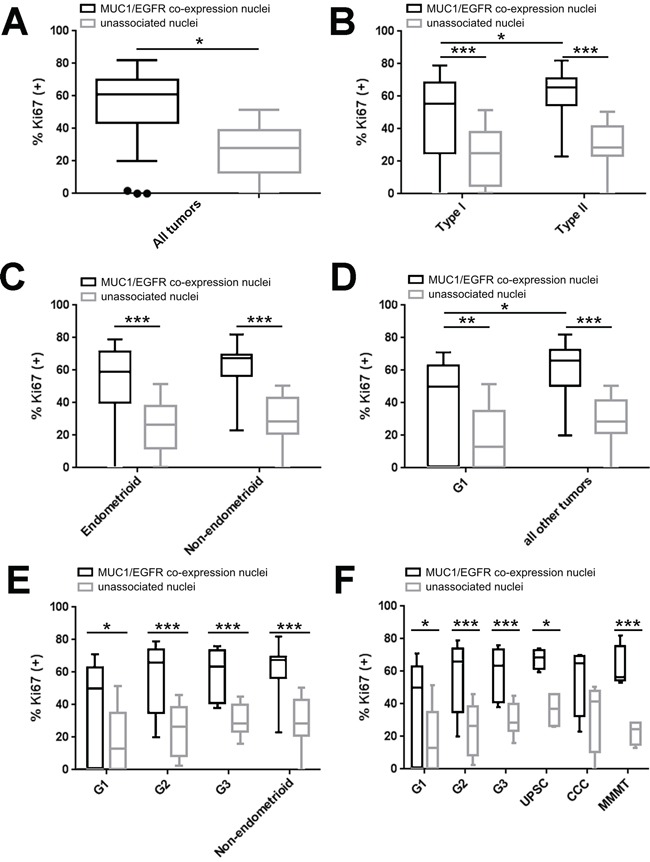
MUC1-EGFR co-expression is associated with higher cellular proliferation in human endometrial tumors Tukey box plots of the ratio of Ki67 positive nuclei in cells associated with MUC1 and EGFR co-expression (black boxes) or of unassociated nuclei (grey boxes). **A.** Comparison of all tumors in aggregate. **B.** Comparison of Type I (grade 1 [G1] and grade 2 [G2] endometrioid endometrial carcinoma [EEC]) with Type II (grade 3 [G3] EEC and all non-endometrioid) tumors. **C.** Comparison of endometrioid and non-endometrioid tumors. **D.** Comparison of G1 EEC *vs*. all other tumors. **E.** Comparison of G1 EEC, G2 EEC, G3 EEC and non-endometrioid tumors. **F.** Comparison of G1 EEC, G2 EEC, G3 EEC, uterine papillary serous carcinoma (UPSC), clear cell carcinoma (CCC) and malignant mixed Mullerian tumor (MMMT). Sidak's multiple comparisons test: p<0.05 (*), p<0.01 (**), p<0.001 (***).

To test the clinical relevance of MUC1-EGFR co-expression, endometrial tumor sections were stained for nuclei, MUC1, EGFR and the proliferation marker, Ki67. Typical staining patterns included EGFR expression without MUC1 (Figure 6A), MUC1 and EGFR expression with little co-expression (Figure 6B) and strong MUC1 and EGFR with high co-expression (Figure 6C). In contrast, MUC1 in normal uterine epithelium was restricted to the apical epithelial surface whereas EGFR expression was low and primarily stromal (Figure 6D). CellProfiler was used to calculate the proportion of each tumor with MUC1 expression, EGFR expression, MUC1/EGFR co-expression as well as the intensity of associated staining. Overall, the percent of endometrial tumor tissue with observed MUC1 expression was 29.5% (± 3.7% SEM), EGFR expression was 40.2% (± 3.9% SEM) and MUC1/EGFR co-expression was 14.3% (± 2.2% SEM). Expression, co-expression or intensity of MUC1 and EGFR did not differ by endometrial cancer grade or histotype (Supplementary Tables 3 and 4). Assessment of cellular proliferation showed an increased proportion of Ki67 positive nuclei associated with MUC1/EGFR co-expression than unassociated nuclei (Figure 7A–7F). This observation was statistically significant in all comparisons between histotypes and grades with the exception of clear cell carcinoma. Furthermore, Type II tumors had significantly higher Ki67 positivity than Type I tumors.

## DISCUSSION

This study shows that MUC1 increases EGFR mRNA and protein expression in endometrial cancer cells, extending observations as reported in other cell types [16]. We used endometrial cancer lines isolated from well- [19], moderately-[20] and poorly-differentiated [21] tumors, suggesting that this may be a general characteristic of MUC1-expressing endometrial cancers. Previously, we reported that activation of EGFR increases MUC1 expression [18]. These data in conjunction with MUC1-driven EGFR expression indicate that MUC1 and EGFR are involved in a co-stimulatory loop. In normal human endometrium, MUC1 and EGFR interactions may be limited to glandular epithelium which display the highest EGFR levels [22, 23]. In endometrial cancer, loss of polarity coupled with elevated expression of both MUC1 and EGFR could enhance the activity of this positive feedback loop [24–27].

For the first time, we have shown that MUC1-Cter stimulates the *EGFR* promoter though direct interaction. This is further supported by the observation that mutation of the CQC motif attenuates stimulation of *EGFR* promoter activity. A small proportion of the MUC1-Cter population is known to translocate to the nucleus through interaction with nucleoprotein Nup62 and Importin β [11]. However, the exact mechanism remains unclear. We identified CCAAT/enhancer binding protein β, p53 and glucocorticoid receptor α as putative MUC1-Cter binding partners on the *EGFR* promoter. Each of these transcription factors are known to associate with MUC1-Cter [8, 9, 28–31]. Further study is required to fully understand the role that these putative transcription factors play in mediating the regulation of *EGFR* by MUC1-Cter.

In accordance with previous reports [16, 32–35], MUC1 increases cellular proliferation, survival and spheroid formation in endometrial cancer cells. As observed in *MUC1* knockdown, pEGFR levels were more strongly reduced than total EGFR levels. This suggests two regulatory mechanisms: 1) MUC1 elevates EGFR levels transcriptionally and 2) MUC1 enhances EGFR signaling. This is further supported by a similar study of non-small cell lung cancer where suppression or inhibition of MUC1 reduced EGFR signaling [36]. MUC1 is reported to directly associate with EGFR through interaction with galectin-3 and to be tyrosine phosphorylated by EGFR at the YEKV motif [37, 38]. However, we were unable to observe a direct interaction between MUC1 and EGFR (data not shown). After activation, EGFR is internalized and degraded to attenuate signaling [39]. MUC1 is linked with inhibition of ligand-directed degradation of EGFR [40]. Thus, in addition to reduced EGFR levels, reduced cell surface EGFR expression may account for attenuated EGFR signaling in the absence of MUC1.

Two EGFR inhibitors have been tested in clinical trials for advanced or recurrent endometrial cancer. Erlotinib showed an objective response rate of 12.5%, with 52.6% of EGFR positive tumors showing a partial response or stable disease [41]. Lapatinib treatment resulted in 26.6% of patients with partial response or stable disease [42]. MUC1-directed therapies have not been tested in endometrial cancer, but there are promising results from MUC1 immunotherapy [43] and ongoing MUC1 inhibitor clinical trials (NCT01279603, NCT02204085). HEC1A cells were sensitized to lapatinib in the absence of MUC1. These data indicate that dual-targeted therapies would be more effective; however, this may be a cell line-specific effect. Interestingly, anti-MUC1 antibodies decrease EGFR signaling and MUC1/EGFR co-inhibition synergistically suppresses cell growth [36, 44]. It would follow that co-targeting of MUC1 and EGFR in endometrial cancer could provide a more effective therapy than targeting EGFR alone.

EGFR polymorphisms do not contribute to poor prognosis in endometrial cancer [45]. Elevated MUC1 or EGFR expression is, however, associated with poor prognosis [13, 15]. This indicates that EGFR levels are the more important prognostic factor. In this study, MUC1-EGFR co-expression was associated with increased cellular proliferation. This was observed in grade 1, grade 2, and grade 3 endometrioid endometrial carcinoma, uterine papillary serous carcinoma and malignant mixed mullerian tumors, but not in clear cell carcinoma tumors. Additionally, increased Ki67 positivity was observed in high grade and Type II tumors, which have poor prognosis [46, 47]. These data recapitulate the *in vitro* observations that MUC1 increases cellular proliferation.

In summary, we demonstrated that MUC1 increases expression and signaling of EGFR. This resulted in increased cellular proliferation, spheroid formation and survival. In addition, MUC1 knockout sensitized cells to the EGFR inhibitor, lapatinib. Finally, MUC1-EGFR co-expression was associated with increased cellular proliferation in human tumors. These data constitute the first evidence that MUC1 stimulates EGFR expression and signaling in endometrial cancer. Future evaluation of advanced endometrial cancer for MUC1 and EGFR expression followed by co-targeting may provide a new avenue for the treatment of advanced endometrial cancer.

## MATERIALS AND METHODS

### Cell culture

HEC1A cells were purchased from the American Type Culture Collection (ATCC, Manassas, VA). Ishikawa cells were kindly provided by Dr. Bruce Lessey (Greenville Health System, SC), and HEC50 cells by Dr. Bryan Hennessy (The University of Texas MD Anderson Cancer Center [MDACC], Houston, TX). Cell lines were maintained at 37°C in a humidified atmosphere of 95% air, 5% CO_2_ (v/v) in DMEM/F12 (Invitrogen, Carlsbad, CA) supplemented with 10% (v/v) fetal bovine serum (FBS; Atlanta Biologicals, Lawrenceville, GA) and 1% (v/v) penicillin-streptomycin solution (Corning Inc., Corning, NY). HEK293 cells (ATCC) were maintained in RPMI (Invitrogen) supplemented with 5% (v/v) FBS and 1% (v/v) penicillin-streptomycin solution.

### Plasmids

A luciferase plasmid containing the *EGFR* −1109/−1 promoter [48] was kindly gifted by Dr. Wenlong Bai from the University of South Florida (Tampa, FL). The pGL3.50, pGL3.10, and pRL-TK plasmids were obtained from Promega (Madison, WI). Tet-inducible Cas9, MUC1 gRNA, pMD2. G and psPAX2 vectors were purchased from the Baylor College of Medicine Cell-Based Assay Screening Service (Houston, TX). The MUC1 gRNA sequences included AAGAAAGGAGACTGGGTGCC targeting the first exon and AGGTGGAGAAAAGGAGACTT targeting the second exon of MUC1. MUC1-Cter and MUC1-Cter-AQA mutant coding sequences were purchased as gBlock gene fragments from Integrated DNA Technologies (Coralville, IA) and cloned using manufacturer's instructions into the pC3 vector backbone (Clontech Laboratories).

### Antibodies

Primary antibodies included rabbit anti-MUC1 C-terminus CT-1 [49], Armenian hamster anti-MUC1 C-terminus CT-2 (MH1, ProSci Inc., Poway, CA), mouse anti-EGFR (H9B4, Thermo Scientific), mouse anti-EGFR (H11, Thermo Scientific), mouse anti-β-actin (ab8226, Abcam, Cambridge, UK), rabbit anti-EGFR-phospho-Y1068 (Invitrogen), rabbit anti-ERK (Cell Signaling Technology, Danvers, MA), rabbit anti-phospho-ERK (E10, Cell Signaling Technology), and rabbit anti-Ki67 (Novus Biologicals Inc., Littleton, CO). Secondary antibodies for western blotting included horseradish peroxidase-conjugated sheep anti-mouse IgG (Jackson ImmunoResearch, West Grove, PA) and horseradish peroxidase-conjugated goat anti-rabbit IgG (Sigma-Aldrich, St. Louis, MO). Secondary antibodies for immunofluorescence included AlexaFluor-488-conjugated goat anti-mouse (Life Technologies, Carlsbad, CA), AlexaFluor-488-conjugated goat anti-rabbit (Life Technologies), AlexaFluor-568-conjugated goat anti-rabbit (Life Technologies), AlexaFluor-647-conjugated goat anti-Armenian hamster (Fisher Scientific), AlexaFluor-647-conjugated goat anti-rabbit (Life Technologies) and AlexaFluor-647-conjugated goat anti-mouse (Life Technologies).

### Transient transfections and reporter assays

Cells were grown in six-well plates as described above, but without antibiotic to ~80% confluence and media replaced with Opti-MEM (Life Technologies). Transfections were performed using Lipofectamine 2000 (Life Technologies) per manufacturer's instructions. Briefly, 500 ng of the reporter plasmid, 500 ng of pRL-TK plasmid (10 ng for HEC50), and 5 uL Lipofectamine 2000 were mixed into 0.5 mL Opti-MEM. Cells were incubated with liposome-DNA complexes for 6-12 h at 37°C. Cells then were washed with 1x PBS and incubated for 23-27 hr in growth media. Cell lysates were collected using the dual-luciferase assay kit (Promega) per manufacturer's instructions. Luciferase was measured using a Tecan Infinite M1000 with injector module (Tecan, Männedorf, Switzerland). Luminescence was expressed as the ratio of firefly luciferase to *Renilla* luciferase and normalized to the expression level of the pGL3.10 control plasmid.

### CRISPR/Cas knockout of MUC1

Lentivirus particles were produced by transient co-transfection of 1 μg of Cas9 or *MUC1* gRNA expression vectors with 250 ng pMD2.G, 750 ng psPAX2 and 6 μL Lipofectamine 2000 into HEK293 cells as described above. Two day conditioned media was filtered through a 0.45 μm filter and 1 mL used to treat HEC1A cells overnight in the presence of 8 μg/mL polybrene (Sigma-Aldrich). Cells then were selected with 500 μg/mL geneticin (VWR, Radnor, PA) and clonal populations isolated. Clones were treated with 2 μM doxycycline (VWR) and screened by western blotting for Cas9 expression. Selected HEC1A-Cas9 clones then were treated with 1 mL MUC1 gRNA viruses in the presence of 8 μg/mL polybrene overnight. Clonal populations of cells then were tested for MUC1 expression *via* immunofluorescence and immunoblotting.

### siRNA knockdown of MUC1

Cells were grown to approximately 30% confluence in 6 well plates (Corning) in growth media without antibiotic. Sixty pmol siRNA targeting *MUC1* [40] or a non-targeted scrambled siRNA (Qiagen, Venlo, Netherlands) were transfected into cells with 3 μL Lipofectamine 2000 and 0.5 mL Opti-MEM (Life Technologies) per well, per manufacturer's instructions. Cells were incubated with liposome-siRNA complexes for 6-12 hr. Cells were then washed with 1x PBS and allowed to recover for 20-30 hr in growth media without antibiotic. A second round of siRNA treatment was then performed for 6-12 hr followed by 20-30 hr of recovery. Cells were then processed for immunoblotting, real-time PCR or luciferase assays. For luciferase assays with *MUC1* knockdown, the luciferase cocktail was added with the second round of siRNA treatment.

### EGF treatment for EGFR phosphorylation assays

Cells were grown to >80% confluence in 6 well plates in growth media, washed 1x with PBS and incubated with DMEM/F12 without serum overnight. Cells then were treated with 10 ng/mL epidermal growth factor (EGF, Sigma-Aldrich) or vehicle (0.1% [w/v] bovine serum albumin [BSA; Sigma-Aldrich]) in PBS, in DMEM/F12 for 5 min. Cells then were lysed and processed for immunoblotting.

### Immunoblotting

Protein was extracted from cells in 6 well plates with 200 μL of sample extraction buffer and mixed 1:1 with Laemmli sample buffer, incubated at 95°C for 5 min and immunoblotted as previously described [18]. Blots were developed using WestDura ECL (Thermo Scientific) as described by the manufacturer and exposed to HyBlot CL autoradiographic film (Denville Scientific Inc., Holliston, MA). Densitometry was analyzed using ImageJ software [50].

### RNA isolation and quantitative PCR

Total RNA was extracted from cells using TRIzol (Invitrogen) and genomic DNA digested using the DNA-free kit (Ambion, Austin, TX) per manufacturer's instructions. Reverse transcription was performed on 2 μg total RNA in a 20 μL volume using qScript Reverse Transcriptase kit (Qiagen) per manufacturer's instructions. Quantitative PCR was performed with 25 μL reactions containing 12.5 μL of SYBR Green PCR master mix (Quanta Biosciences, Inc., Gaithersburg, MD), 1 μL forward primer, 1 μL reverse primer, 2 μL cDNA and 8.5 μL nuclease free water (Fisher Scientific). All primers were purchased from Integrated DNA Technologies (Coralville, IA). All primers and cycle conditions can be found in Supplementary Table 1. Reactions were performed with a CFX96 Real-Time PCR Detection system (Bio-Rad Laboratories, Inc., Hercules, CA). Relative amounts of mRNA were calculated using the ΔΔCt method [51].

### Chromatin immunoprecipitation

ChIP of HEC50 cells was performed using the Chromatrap® ChIP Kit (Porvair Sciences, Norfolk, UK) per manufacturer's instructions. Briefly, cells were grown in 75 cm^2^ flasks to confluence and then fixed with 1% (w/v) formaldehyde (Fisher Scientific) in DMEM/F12 for 10 min, followed by one wash using ice cold PBS. Fixation quenched with 0.65 M glycine for 15 min. Cells then were treated with 0.05% (v/v) Trypsin-EDTA solution (Life Technologies) for 10 min, washed with ice cold PBS, and scraped into 10 ml PBS containing 1 mM phenylmethylsulfonyl fluoride (Sigma-Aldrich). This suspension was centrifuged for 5 min at 1000 rpm. Cell pellets were frozen and stored at −80°C with 1 μL protease inhibitor cocktail solution (EMD Millipore). Pellets were resuspended in 1 mL lysis buffer and sheared using a Bioruptor® (Diagenode, Denville, NJ) to a size of 200-500 bp. ChIP was performed with 10 μg chromatin and 20 μg anti-MUC1-Cter antibody (CT-1) and processed according to Chromatrap® protocol version 6. HEC1A ChIP was performed using the Re-ChIP-IT kit (Active Motif, Carlsbad, CA) per manufacturer's instructions. Chromatin was prepared as described above with the exception of using a Cell Disruptor Tip Sonicator (Ultrasonics, Inc.) for shearing. The ChIP reaction contained 25 μL protein G magnetic beads, 15 μg chromatin and 7 μg anti-MUC1-Cter antibody (CT-2). Chromatin was eluted with 50 μL Elution Buffer AM2 and reverse crosslinked overnight at 65°C. Quantitative PCR was performed using 20 μL reaction volumes containing 10 μL iQ SYBR Green Supermix (BioRad), 1 μL forward primer, 1 μL reverse primer, and 8 μL ChIP enriched DNA. Primer sets and cycle conditions can be found in Supplementary Table 1. Reactions were performed with a CFX96 Real-Time PCR Detection system. Data was normalized to input chromatin samples.

### Immunofluorescence

All patient samples were collected as described previously [52] at MDACC and processed at Rice University under approval of Institutional Review Boards from each institution. Endometrial tumor grade and histotype can be found in Supplementary Table 2. Cells were grown in Nunc Lab-Tek II chambered glass slides (Sigma-Aldrich) to ~80% confluence. Cells and tissues were fixed with 4% (w/v) paraformaldehyde (Fisher Scientific) in PBS for 10 min, permeabilized with 0.25% (v/v) triton X-100 in PBS for 2 min and then blocked with 1% (w/v) BSA in PBS for 1 hr. This was followed by incubation in a 1:200 dilution of primary antibody in blocking buffer for 1 hr and then a 1:400 dilutions of secondary antibody in blocking buffer. Fixing, permeabilization and antibody incubation steps were followed by a 3 × 5 min wash with PBS. Slides then were sealed with ProLong Gold Antifade with DAPI (Life Technologies) and a coverslip. All slides were imaged with a Nikon A1-Rsi confocal microscope (Nikon, Tokyo, Japan) or a Zeiss LSM 710 confocal (Carl Zeiss AG, Jena, Germany).

### Analysis of endometrial tumor sections

Immunofluorescence images of endometrial tumors were processed using CellProfiler [53]. Protein expression was defined as pixels with intensity exceeding 0.02 intensity units. Detected expression of MUC1 and EGFR was expanded by 5 pixels for membrane expression to overlap nuclei and subsequently defined as MUC1 and EGFR expression masks. Overlap of the MUC1 and the EGFR mask defined MUC1+EGFR co-expression and the co-expression mask. The ratio of the area of each mask to the total tumor area (area of pixels in all channels with intensity > 0.02) defined the percent of tumor positive for each expression pattern. The integrated pixel intensity of the signaling from MUC1 alone, EGFR alone, or MUC1+EGFR co-expression was then calculated. The ratio of the integrated pixel intensity to percent of tumor positive for each mask was calculated to describe the intensity per percent of tumor stained. Nuclei were then associated with expression patterns described by detecting overlap with each mask, inclusive of partially overlapping nuclei. Unassociated nuclei included nuclei lacking overlap with any mask. The ratio of Ki67 positive nuclei was then calculated in each case.

### MTS assays

Before seeding, cells were washed 3x with 5 mL DMEM/F12. Then, 20,000 cells were seeded per well into 96 well plates containing 200 μL DMEM/F12 with 50 ng/mL EGF or vehicle (0.1% [w/v] BSA in PBS). CellTiter 96 AQueous One Solution Cell Proliferation Assay (Promega) was then performed per manufacturer's instructions. Absorbance was read with a Tecan M1000 plate reader (Tecan Group Ltd., Mannedorf, Switzerland). Background absorbance (culture media without cells) was subtracted, and then values were normalized to day 0.

### Three dimensional spheroid formation and analysis

HyStem® hydrogels (ESI Bio, Alameda, CA) were reconstituted and mixed per manufacturer's instructions. Before seeding, cells were washed 3x with 5 mL DMEM/F12. Next, 50,000 cells were added per 20 μL of a 1:4 ratio of Extralink® to HyStem® and then 20 μL was dispensed per well into Aurora 384 well plates (Brooks Automation, Inc., Chelmsford, MA). After 30 min of crosslinking, 50 μL of DMEM/F12 with 50 ng/mL EGF or vehicle (0.1% [w/v] BSA in PBS) was added per well. After 3 and 5 days, media was replaced with 1x PBS containing 4 μM calcein AM (Life Technologies), 4 μM ethidium homodimer-1 (Life Technologies), and 4 μM bisBenzimide H 33342 trihydrochloride (Sigma-Aldrich). Cells then were incubated for 1 hr, and imaged with a Nikon A1-Rsi confocal. Resultant images were analyzed for cell survival, spheroid counts and spheroid diameter as previously described [54].

### EGFR inhibitor treatments

Erlotinib and AG490 were purchased from Cayman Chemical Company (Ann Arbor, MI) and EMD Millipore, respectively. Lapatinib was obtained from the NIH drug repository. 20,000 cells were seeded per well into 96 well plates in DMEM/F12 + 10% (v/v) FBS. After one day, media was replaced with DMEM/F12 + 10% (v/v) FBS containing 0, 1, 10 or 20 μM drug. On days 0 and 3 of drug treatment, MTS was performed as described above.

### Statistical analysis

All bar graphs represent means ± one standard deviation of at least triplicate samples and are representative of at least two independent experiments. All box plots are Tukey box plots with outliers as dots. Quantitative PCR, densitometry, luciferase and MTS were analyzed using unpaired two-tailed Student's T-test. Three dimensional culture data was analyzed by one-way ANOVA with Tukey post-test. Endometrial tumor expression data was compared with an unpaired, two-tailed Student's t test in pairwise comparisons, or one-way ANOVA with Tukey's post-test for larger groups. Data Ki67 positivity was analyzed using matched, two-way ANOVA with Tukey's or Sidak's post-test (factor A: tumor, factor B: Ki67). All statistical analyses were performed with GraphPad InStat (GraphPad Software, San Diego, CA).

## SUPPLEMENTARY FIGURES AND TABLES




