# Supplementary material for: MUC1 stimulates EGFR expression and function in endometrial cancer
Source: Oncotarget. 2016 Apr 15;7(22):32796–809. doi: 10.18632/oncotarget.8743 (PMC5078052; doi:10.18632/oncotarget.8743)
Supplement: Supplementary file 1 [file oncotarget-07-32796-s001.pdf]

MUC1 stimulates EGFR expression and function in endometrial cancer

SUPPLEMENTARY FIGURE AND TABLES

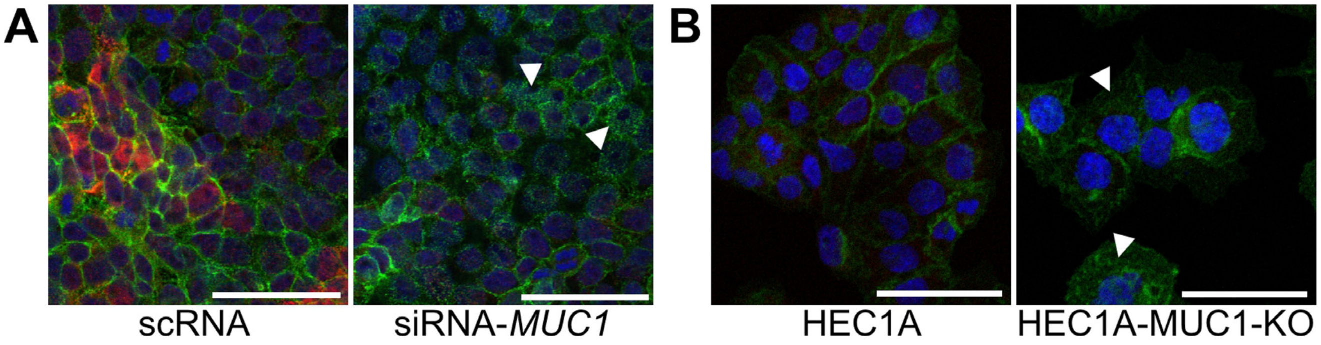

Supplementary Figure 1: MUC1 expression is associated with punctate EGFR expression. A. HEC50 cells were pretreated with siRNA-*MUC1* or scRNA and then immunostained for MUC1 (red), EGFR (green) and nuclei (blue). **B.** HEC1A and HEC1A-MUC1-KO cells were immunostained for MUC1 (red), EGFR (green) and nuclei (blue). In both cases, the absence of MUC1 results in punctate EGFR staining, indicative of vesicular localization (white arrowheads). Scale bars represent 50 µm.

Supplementary Table 1: PCR primer sequences and cycle conditions

Target	Forward Primer	Reverse Primer	Cycle Conditions
β-actin	5’-GATGAGATTGGCATGGCTTT-3’	5’-CACCTTCACCGGTCCAGTTT-3’	95°C 2 min, [95 °C 15 sec, 60°C 30 sec] x39 cycles
MUC1	5’-GTGCCCCCTAGCAGTACCG-3’	5’-GACGTGCCCCTACAAGTTGG-3’	95°C 2 min, [95 °C 15 sec, 60°C 30 sec] x39 cycles
EGFR	5’-TCCCTCAGCCACCCATATGTA-3’	5’GTCTCGGGGCATTTTGGAGAA-3’	[95°C 30 sec, 55°C 30 sec, 72°C 30 sec] x39 cycles
EGFR promoter -1109/-985	5’-AGGGGCAGTGGGACACTTAG-3’	5’-CATGGGTACTTTGAAGCCAATGTG-3’	95°C 2 min, [95 °C 30 sec, 58°C 1 min, 72°C 30 sec] x39 cycles
EGFR promoter -627/-511	5’-GCACAGATTTGGCTCGACCTGGA-3’	5’-GAGCGGGTGCCCTGAGGAGTTAATT-3’	95°C 2 min, [95 °C 15 sec, 58°C 30 sec, 72°C 30 sec] x39 cycles
EGFR promoter -486/-374	5’-CGCCGGAGACTAGGTCCC-3’	5’-AGGAGGAGGAGAATGCGAGGA-3’	95°C 2 min, [95 °C 15 sec, 58°C 30 sec, 72°C 30 sec] x39 cycles
EGFR promoter -296/-198	5’-TAGACGTCCGGGCAGCCCCC-3’	5’-TCGGGACTCCGGCCGCCT-3’	95°C 2 min, [95 °C 15 sec, 58°C 30 sec, 72°C 30 sec] x39 cycles
EGFR promoter -172/-64	5’-AGACCGGACGACAGGCCACCT-3’	5’-TCCCGATCAATACTGGACGGAGTCAG-3’	95°C 2 min, [95 °C 15 sec, 58°C 30 sec, 72°C 30 sec] x39 cycles

Supplementary Table 2: Endometrial carcinomas by grade and histotype

Endometrial Carcinomas	(n=)
G1 EEC	9
G2 EEC	9
G3 EEC	8
UPSC	4
CCC	4
MMMT	5

Abbreviations: G1 EEC, grade 1 endometrioid endometrial carcinoma; G2 EEC, grade 2 endometrioid endometrial carcinoma; G3 EEC, grade 3 endometrioid endometrial carcinoma; UPSC, uterine papillary serous carcinoma; CCC, clear cell carcinoma; MMMT, malignant mixed mullerian tumor (Uterine Carcinosarcoma)

Supplementary Table 3: Statistical comparisons of MUC1 and EGFR expression in human endometrial tumors

Abbreviations: G1 EEC, grade 1 endometrioid endometrial carcinoma; G2 EEC, grade 2 endometrioid endometrial carcinoma; G3 EEC, grade 3 endometrioid endometrial carcinoma; UPSC, uterine papillary serous carcinoma; CCC, clear cell carcinoma; MMMT, malignant mixed mullerian tumor (Uterine Carcinosarcoma)

Classifications: Endometrioid Grade (G1 EEC) vs. (G2 EEC) vs. (G3 EEC) vs. Non-Endometrioid (UPSC + CCC + MMMT); Endometrioid (G1 EEC + G2 EEC + G3 EEC) vs. Non-Endometrioid (UPSC + CCC + MMMT); Type I (G1 EEC + G2 EEC) vs. Type II (UPSC + CCC + MMMT)

See Supplementary File 1

Supplementary Table 4: Statistical comparisons of MUC1 and EGFR expression in human endometrial tumors excluding MMMT (uterine carcinosarcoma)

Abbreviations: G1 EEC, grade 1 endometrioid endometrial carcinoma; G2 EEC, grade 2 endometrioid endometrial carcinoma; G3 EEC, grade 3 endometrioid endometrial carcinoma; UPSC, uterine papillary serous carcinoma; CCC, clear cell carcinoma.

Classifications: Endometrioid Grade (G1 EEC) vs. (G2 EEC) vs. (G3 EEC) vs. Non-Endometrioid (UPSC + CCC); Endometrioid (G1 EEC + G2 EEC + G3 EEC) vs. Non-Endometrioid (UPSC + CCC); Type I (G1 EEC + G2 EEC) vs. Type II (UPSC + CCC)

See Supplementary File 1
