# Supplementary material for: MUC1 stimulates EGFR expression and function in endometrial cancer
Source: Oncotarget. 2016 Apr 15;7(22):32796–809. doi: 10.18632/oncotarget.8743 (PMC5078052; doi:10.18632/oncotarget.8743)
Supplement: Supplementary file 2 [file oncotarget-07-32796-s002.docx]

**Supplementary Table 3. Statistical comparisons of MUC1 and EGFR expression in human endometrial tumors.**

| **Grade &**  **Histotype** | % of  tissue with MUC1 expression | % of  tissue with EGFR expression | % of  tissue with MUC1/EGFR co-expression | MUC1 intensity per % of tissue | MUC1 intensity in co-expression per % of tissue | EGFR intensity per % of tissue | EGFR intensity in co-expression per % of tissue |
| --- | --- | --- | --- | --- | --- | --- | --- |
| G1 EEC | 18.90 ± 5.477 | 31.53 ± 7.495 | 7.221 ± 2.151 | 1210.0 ± 441.4 | 88.17 ± 17.15 | 154.60 ± 5.929 | 66.57 ± 35.04 |
| G2 EEC | 30.37 ± 5.647 | 43.95 ± 8.749 | 14.64 ± 3.199 | 605.50 ± 67.33 | 54.20 ± 4.977 | 175.00 ± 17.19 | 31.11 ± 8.113 |
| G3 EEC | 31.61 ± 10.39 | 45.01 ± 9.041 | 14.53 ± 6.699 | 619.70 ± 91.46 | 61.50 ± 18.42 | 155.90 ± 19.89 | 42.21 ± 15.51 |
| UPSC | 42.64 ± 5.852 | 31.75 ± 7.814 | 17.91 ± 6.983 | 498.40 ± 16.42 | 62.72 ± 14.41 | 125.70 ± 12.06 | 10.32 ± 3.600 |
| CCC | 47.62 ± 18.61 | 48.20 ± 13.41 | 24.27 ± 8.183 | 579.40 ± 36.14 | 52.95 ± 16.41 | 179.90 ± 38.06 | 23.27 ± 12.97 |
| MMMT | 18.28 ± 10.17 | 41.37 ± 13.40 | 15.03 ± 8.541 | 1331.0 ± 503.10 | 65.38 ± 33.15 | 183.70 ± 60.69 | 168.80 ± 114.00 |
| ***P=*** | **0.2429** | **0.7798** | **0.4749** | **0.2708** | **0.6839** | **0.7501** | **0.2136** |

| **Endometrioid Grade**  **vs.**  **Non-Endometrioid** | % of  tissue with MUC1 expression | % of  tissue with EGFR expression | % of  tissue with MUC1/EGFR co-expression | MUC1 intensity per % of tissue | MUC1 intensity in co-expression per % of tissue | EGFR intensity per % of tissue | EGFR intensity in co-expression per % of tissue |
| --- | --- | --- | --- | --- | --- | --- | --- |
| G1 EEC | 18.90 ± 5.477 | 31.53 ± 7.495 | 7.221 ± 2.151 | 1210.00 ± 441.4 | 88.17 ± 17.15 | 154.60 ± 5.929 | 66.57 ± 35.04 |
| G2 EEC | 30.37 ± 5.647 | 43.95 ± 8.749 | 14.64 ± 3.199 | 605.50 ± 67.33 | 54.20 ± 4.977 | 175.00 ± 17.19 | 31.11 ± 8.113 |
| G3 EEC | 31.61 ± 10.39 | 45.01 ± 9.041 | 14.53 ± 6.699 | 619.70 ± 91.46 | 61.50 ± 18.42 | 155.90 ± 19.89 | 42.21 ± 15.51 |
| Non-Endometrioid | 34.80 ± 7.555 | 40.51 ± 6.713 | 18.76 ± 4.416 | 843.40 ± 212.2 | 60.74 ± 13.41 | 164.70 ± 25.52 | 75.25 ± 46.23 |
| ***P=*** | **0.4744** | **0.6544** | **0.3056** | **0.3520** | **0.3900** | **0.9040** | **0.7948** |

| **Endometrioid**  **vs.**  **Non-Endometrioid** | % of  tissue with MUC1 expression | % of  tissue with EGFR expression | % of  tissue with MUC1/EGFR co-expression | MUC1 intensity per % of tissue | MUC1 intensity in co-expression per % of tissue | EGFR intensity per % of tissue | EGFR intensity in co-expression per % of tissue |
| --- | --- | --- | --- | --- | --- | --- | --- |
| Endometrioid | 26.78 ± 4.181 | 39.98 ± 4.823 | 12.04 ± 2.450 | 819.00 ± 161.40 | 68.21 ± 8.563 | 162.00 ± 8.620 | 46.80 ± 13.16 |
| Non-Endometrioid | 34.80 ± 7.555 | 40.51 ± 6.713 | 18.76 ± 4.416 | 843.40 ± 212.20 | 60.74 ± 13.41 | 164.70 ± 25.52 | 75.25 ± 46.23 |
| ***P=*** | **0.3194** | **0.9497** | **0.1569** | **0.9292** | **0.6299** | **0.9024** | **0.4504** |

| **Type I**  **vs.**  **Type II** | % of  tissue with MUC1 expression | % of  tissue with EGFR expression | % of  tissue with MUC1/EGFR co-expression | MUC1 intensity per % of tissue | MUC1 intensity in co-expression per % of tissue | EGFR intensity per % of tissue | EGFR intensity in co-expression per % of tissue |
| --- | --- | --- | --- | --- | --- | --- | --- |
| Type I | 24.63 ± 4.061 | 37.74 ± 5.788 | 10.93 ± 2.075 | 907.60 ± 228.60 | 71.19 ± 9.592 | 164.80 ± 9.163 | 48.84 ± 17.97 |
| Type II | 33.58 ± 5.976 | 42.22 ± 5.280 | 17.15 ± 3.666 | 758.20 ± 135.80 | 61.03 ± 10.58 | 161.30 ± 17.19 | 62.66 ± 28.96 |
| ***P=*** | **0.2388** | **0.5704** | **0.1663** | **0.5649** | **0.4875** | **0.8674** | **0.6991** |

| **G1 EEC**  **vs.**  **all other**  **tumors** | % of  tissue with MUC1 expression | % of  tissue with EGFR expression | % of  tissue with MUC1/EGFR co-expression | MUC1 intensity per % of tissue | MUC1 intensity in co-expression per % of tissue | EGFR intensity per % of tissue | EGFR intensity in co-expression per % of tissue |
| --- | --- | --- | --- | --- | --- | --- | --- |
| G1 EEC | 18.90 ± 5.477 | 31.53 ± 7.495 | 7.221 ± 2.151 | 1210.00 ± 441.4 | 88.17 ± 17.15 | 154.60 ± 5.929 | 66.57 ± 35.04 |
| all other  tumors | 32.62 ± 4.467 | 42.74 ± 4.451 | 16.40 ± 2.717 | 712.40 ± 97.17 | 58.98 ± 7.515 | 165.40 ± 12.98 | 53.20 ± 20.44 |
| ***P=*** | **0.1245** | **0.2264** | **0.0821** | **0.0998** | **0.0863** | **0.6553** | **0.7519** |

Abbreviations: G1 EEC, grade 1 endometrioid endometrial carcinoma; G2 EEC, grade 2 endometrioid endometrial carcinoma; G3 EEC, grade 3 endometrioid endometrial carcinoma; UPSC, uterine papillary serous carcinoma; CCC, clear cell carcinoma; MMMT, malignant mixed mullerian tumor (Uterine Carcinosarcoma)

Classifications: Endometrioid Grade (G1 EEC) vs. (G2 EEC) vs. (G3 EEC) vs. Non-Endometrioid (UPSC + CCC + MMMT); Endometrioid (G1 EEC + G2 EEC + G3 EEC) vs. Non-Endometrioid (UPSC + CCC + MMMT); Type I (G1 EEC + G2 EEC) vs. Type II (UPSC + CCC + MMMT)

**Supplementary Table 4. Statistical comparisons of MUC1 and EGFR expression in human endometrial tumors** **excluding MMMT (uterine carcinosarcoma).**

| **Grade &**  **Histotype** | % of  tissue with MUC1 expression | % of  tissue with EGFR expression | % of  tissue with MUC1/EGFR co-expression | MUC1 intensity per % of tissue | MUC1 intensity in co-expression per % of tissue | EGFR intensity per % of tissue | EGFR intensity in co-expression per % of tissue |
| --- | --- | --- | --- | --- | --- | --- | --- |
| G1 EEC | 18.90 ± 5.477 | 31.53 ± 7.495 | 7.221 ± 2.151 | 1210.0 ± 441.4 | 88.17 ± 17.15 | 154.60 ± 5.929 | 66.57 ± 35.04 |
| G2 EEC | 30.37 ± 5.647 | 43.95 ± 8.749 | 14.64 ± 3.199 | 605.50 ± 67.33 | 54.20 ± 4.977 | 175.00 ± 17.19 | 31.11 ± 8.113 |
| G3 EEC | 31.61 ± 10.39 | 45.01 ± 9.041 | 14.53 ± 6.699 | 619.70 ± 91.46 | 61.50 ± 18.42 | 155.90 ± 19.89 | 42.21 ± 15.51 |
| UPSC | 42.64 ± 5.852 | 31.75 ± 7.814 | 17.91 ± 6.983 | 498.40 ± 16.42 | 62.72 ± 14.41 | 125.70 ± 12.06 | 10.32 ± 3.600 |
| CCC | 47.62 ± 18.61 | 48.20 ± 13.41 | 24.27 ± 8.183 | 579.40 ± 36.14 | 52.95 ± 16.41 | 179.90 ± 38.06 | 23.27 ± 12.97 |
| ***P=*** | **0.2493** | **0.6309** | **0.2878** | **0.3051** | **0.4244** | **0.4387** | **0.7830** |

| **Endometrioid Grade**  **vs.**  **Non-Endometrioid** | % of  tissue with MUC1 expression | % of  tissue with EGFR expression | % of  tissue with MUC1/EGFR co-expression | MUC1 intensity per % of tissue | MUC1 intensity in co-expression per % of tissue | EGFR intensity per % of tissue | EGFR intensity in co-expression per % of tissue |
| --- | --- | --- | --- | --- | --- | --- | --- |
| G1 EEC | 18.90 ± 5.477 | 31.53 ± 7.495 | 7.221 ± 2.151 | 1210.0 ± 441.4 | 88.17 ± 17.15 | 154.60 ± 5.929 | 66.57 ± 35.04 |
| G2 EEC | 30.37 ± 5.647 | 43.95 ± 8.749 | 14.64 ± 3.199 | 605.50 ± 67.33 | 54.20 ± 4.977 | 175.00 ± 17.19 | 31.11 ± 8.113 |
| G3 EEC | 31.61 ± 10.39 | 45.01 ± 9.041 | 14.53 ± 6.699 | 619.70 ± 91.46 | 61.50 ± 18.42 | 155.90 ± 19.89 | 42.21 ± 15.51 |
| Non-Endometrioid | 45.13 ± 9.081 | 39.97 ± 7.829 | 21.09 ± 5.123 | 538.90 ± 23.93 | 57.84 ± 10.28 | 152.80 ± 21.13 | 16.79 ± 6.693 |
| ***P=*** | **0.1457** | **0.6442** | **0.2044** | **0.1802** | **0.2826** | **0.7546** | **0.3866** |

| **Endometrioid**  **vs.**  **Non-Endometrioid** | % of  tissue with MUC1 expression | % of  tissue with EGFR expression | % of  tissue with MUC1/EGFR co-expression | MUC1 intensity per % of tissue | MUC1 intensity in co-expression per % of tissue | EGFR intensity per % of tissue | EGFR intensity in co-expression per % of tissue |
| --- | --- | --- | --- | --- | --- | --- | --- |
| Endometrioid | 26.78 ± 4.181 | 39.98 ± 4.823 | 12.04 ± 2.450 | 819.00 ± 161.40 | 68.21 ± 8.563 | 162.00 ± 8.620 | 46.80 ± 13.16 |
| Non-Endometrioid | 45.13 ± 9.081 | 39.97 ± 7.829 | 21.09 ± 5.123 | 538.90 ± 23.93 | 57.84 ± 10.28 | 152.80 ± 21.13 | 16.79 ± 6.693 |
| ***P=*** | **0.0506** | **0.9993** | **0.0936** | **0.3482** | **0.5353** | **0.6372** | **0.2247** |

| **Type I**  **vs.**  **Type II** | % of  tissue with MUC1 expression | % of  tissue with EGFR expression | % of  tissue with MUC1/EGFR co-expression | MUC1 intensity per % of tissue | MUC1 intensity in co-expression per % of tissue | EGFR intensity per % of tissue | EGFR intensity in co-expression per % of tissue |
| --- | --- | --- | --- | --- | --- | --- | --- |
| Type I | 24.63 ± 4.061 | 37.74 ± 5.788 | 10.93 ± 2.075 | 907.60 ± 228.60 | 71.19 ± 9.592 | 164.80 ± 9.163 | 48.84 ± 17.97 |
| Type II | 38.37 ± 6.891 | 42.49 ± 5.814 | 17.81 ± 4.161 | 579.30 ± 46.84 | 59.67 ± 10.20 | 154.40 ± 14.03 | 29.50 ± 8.795 |
| ***P=*** | **0.0874** | **0.5680** | **0.1355** | **0.1929** | **0.4167** | **0.5293** | **0.3596** |

| **G1 EEC**  **vs.**  **all other**  **tumors** | % of  tissue with MUC1 expression | % of  tissue with EGFR expression | % of  tissue with MUC1/EGFR co-expression | MUC1 intensity per % of tissue | MUC1 intensity in co-expression per % of tissue | EGFR intensity per % of tissue | EGFR intensity in co-expression per % of tissue |
| --- | --- | --- | --- | --- | --- | --- | --- |
| G1 EEC | 18.90 ± 5.477 | 31.53 ± 7.495 | 7.221 ± 2.151 | 1210.00 ± 441.4 | 88.17 ± 17.15 | 154.60 ± 5.929 | 66.57 ± 35.04 |
| all other  tumors | 35.49 ± 4.841 | 43.02 ± 4.767 | 16.67 ± 2.872 | 588.70 ± 37.79 | 57.70 ± 6.697 | 161.80 ± 10.87 | 30.08 ± 6.234 |
| ***P=*** | **0.0671** | **0.2178** | **0.0676** | **0.0255** | **0.0516** | **0.7015** | **0.1219** |

Abbreviations: G1 EEC, grade 1 endometrioid endometrial carcinoma; G2 EEC, grade 2 endometrioid endometrial carcinoma; G3 EEC, grade 3 endometrioid endometrial carcinoma; UPSC, uterine papillary serous carcinoma; CCC, clear cell carcinoma.

Classifications: Endometrioid Grade (G1 EEC) vs. (G2 EEC) vs. (G3 EEC) vs. Non-Endometrioid (UPSC + CCC); Endometrioid (G1 EEC + G2 EEC + G3 EEC) vs. Non-Endometrioid (UPSC + CCC); Type I (G1 EEC + G2 EEC) vs. Type II (UPSC + CCC)
